# Supplementary material for: Clarifying How Degree Entropies and Degree-Degree Correlations Relate to Network Robustness
Source: Entropy (Basel). 2022 Aug 24;24(9):1182. doi: 10.3390/e24091182 (PMC9497962; doi:10.3390/e24091182)
Supplement: Supplementary file 1 [file entropy-24-01182-s001.zip › entropy-1865693-supplementary.pdf]

Clarifying How Degree Entropies and Degree-Degree Correlations Relate to Network Robustness: Supplementary Materials

1 Network Data

In Table S1, we provide data for 89 different real world networks. The format for each entry in the table is the name of the network, the network's degree distribution, the network's Molloy-Reed critical fraction, and a citation for its source. References for each network's original source is given where possible. All network data was accessed via either The KONECT Project [1] or via Network Repository [2].

Table S1: Real world network data for comparing entropy and crit-ical fraction.

Network Name	$H(p)$	f_c	Reference
air-traffic	2.8931	0.8433	[1]
amazon-links	0.5999	0.9927	[3]
bible-names	4.3568	0.9754	[1]
bio-CE-CX	6.2090	0.9895	[4]
bio-CE-GN	6.8178	0.9891	[4]
bio-CE-GT	3.8530	0.9649	[4]
bio-CE-HT	2.3282	0.7572	[4]
bio-CE-LC	2.2872	0.8717	[4]
bio-CE-PG	6.7551	0.9936	[4]
bio-DM-CX	6.4881	0.9892	[4]
bio-DM-HT	2.8417	0.8177	[4]
bio-dmela-large	3.8786	0.9560	[5]
bio-fly-brain	4.3156	0.9873	[6]
bio-HS-CX	6.8313	0.9918	[4]
bio-HS-HT	4.5671	0.9707	[4]
bio-HS-LC	5.2002	0.9867	[4]
bio-human-protein-large	3.1714	0.9819	[7]
bio-mouse-brain	2.4688	0.9346	[6]
bio-SC-CC	6.1743	0.9886	[4]
bio-SC-GT	6.4340	0.9893	[4]
bio-SC-HT	6.8070	0.9943	[4]

bio-SC-LC	5.5558	0.9801	[4]
bio-yeast-large	2.4519	0.8394	[8]
bio-yeast-small	2.4061	0.8368	[8]
Brightkite_edges	3.6976	0.9841	[9]
ca-CondMat	4.3679	0.9534	[10]
ca-Erdos992	1.9042	0.9353	[11]
ca-GrQc	3.8379	0.9411	[10]
chess	5.1962	0.9785	[1]
cit-citeseer	2.9369	0.8586	[12]
cit-cora	3.0936	0.9024	[13]
cit-DBLP	3.8105	0.9766	[14]
cit-HepPh	5.9810	0.9840	[10]
cit-HepTh	5.9986	0.9905	[10]
cit-iui	3.3176	0.8811	[15]
crime	2.9960	0.8047	[1]
deezer_europe	3.9901	0.9341	[16]
djavax	4.1993	0.9631	[17]
email-Enron	4.0486	0.9929	[18]
email-EU	1.4079	0.9901	[10]
euroroad	2.0037	0.5207	[19]
facebook_combined	6.7701	0.9905	[20]
fb-pages-company	4.1505	0.9525	[21]
fb-pages-government	5.9665	0.9873	[21]
fb-pages-politician	5.0901	0.9760	[21]
fb-pages-public-figure	4.6336	0.9799	[21]
fb-pages-sport	4.9483	0.9733	[21]
fb-pages-tvshow	4.3561	0.9610	[21]
Franz1	1.8412	0.8300	[2]
ia-email-univ	4.5849	0.9435	[22]
ia-fb-messages	4.5414	0.9621	[23]
ia-reality	0.6145	0.9846	[24]
internet	3.7287	0.9729	[25]
lastfm_asia	4.0318	0.9591	[16]
mammalia-voles-bhp-trapping	3.7551	0.8877	[26]
mammalia-voles-kcs-trapping	3.8593	0.8945	[26]
mammalia-voles-plj-trapping	3.9562	0.8965	[26]
mammalia-voles-rob-trapping	3.7053	0.8745	[26]
openflights	4.1541	0.9818	[27]
p2p-Gnutella04	3.9593	0.9229	[28]
p2p-Gnutella05	3.9081	0.9275	[28]
p2p-Gnutella06	3.9110	0.9254	[28]
p2p-Gnutella08	3.6585	0.9400	[28]
p2p-Gnutella09	3.5932	0.9368	[28]

p2p-Gnutella24	3.1667	0.9094	[28]
p2p-Gnutella25	3.1407	0.8974	[28]
phonecalls.edgelist	2.9857	0.7945	[29]
polblogs	5.8433	0.9875	[30]
power-bcspwr10	2.3304	0.7791	[31]
power-US-Grid	2.4552	0.6517	[32]
small	3.4078	0.8513	[2]
soc-advogato	5.1394	0.9873	[33]
soc-anybeat	3.1306	0.9974	[34]
soc-hamsterster	5.2673	0.9766	[1]
soc-sign-bitcoinalpha	3.5773	0.9834	[35]
soc-sign-bitcoinotc	3.4776	0.9873	[35]
soc-wiki-Vote	3.9007	0.9416	[36]
tech-internet-as	2.4294	0.9970	[37]
tech-pgp	3.1474	0.9441	[38]
tech-routers-rf	3.6790	0.9515	[39]
uc-forum	5.1931	0.9712	[40]
uk	0.6803	0.4717	[41]
unicode-language	2.7732	0.9501	[1]
web-edu	1.6571	0.9416	[42]
web-EPA	2.8274	0.9634	[43]
web-indochina-2004	3.8668	0.9572	[44]
web-spam	4.9714	0.9860	[45]
web-webbase-2001	2.1411	0.9899	[44]
wikiquote-be	1.9847	0.9868	[1]

References

[1] J. Kunegis, "Konect: The koblenz network collection," in *Proceedings of the 22nd International Conference on World Wide Web*, p. 1343–1350, Association for Computing Machinery, 2013. http://konect.cc/, accessed 16/05/22.

[2] R. A. Rossi and N. K. Ahmed, "The network data repository with interactive graph analytics and visualization," in *AAAI*, 2015. https://networkrepository.com, accessed 16/05/22.

[3] L. Šubelj and M. Bajec, "Ubiquitousness of link-density and link-pattern communities in real-world networks," *The European Physical Journal B - Condensed Matter and Complex Systems*, vol. 85, 04 2011.

[4] A. Cho, J. Shin, S. Hwang, C. Kim, H. Shim, H. Kim, H. Kim, and I. Lee, "WormNet v3: a network-assisted hypothesis-generating server for Caenorhabditis elegans," *Nucleic Acids Research*, vol. 42, pp. W76–W82, 05 2014.

[5] R. Singh, J. Xu, and B. Berger, "Global alignment of multiple protein interaction networks with application to functional orthology detection," *Proceedings of the National Academy of Sciences*, vol. 105, no. 35, pp. 12763–12768, 2008.

[6] K. Amunts, C. Lepage, L. Borgeat, H. Mohlberg, T. Dickscheid, M.-Rousseau, S. Bludau, P.-L. Bazin, L. Lewis, A.-M. Oros-Peusquens, N. Shah, T. Lippert, K. Zilles, and A. Evans, "Bigbrain: An ultrahigh-resolution 3d human brain model," *Science (New York, N.Y.)*, vol. 340, pp. 1472–1475, 06 2013.

[7] R. M. Ewing, P. Chu, F. Elisma, H. Li, P. J. Taylor, S. Climie, L. D. B. McBroom-Cerajewski, M. D. Robinson, L. O'Connor, M. Li, R. Taylor, M. Dharsee, Y. Ho, A. M. Heilbut, L. Moore, S. Zhang, O. I. Ornatsky, Y. V. Bukhman, M. Ethier, Y. Sheng, J. Vasilescu, M. Abu-Farha, J.-P. Lambert, H. Duewel, I. I. Stewart, B. L. Kuehl, K. A. Hogue, K. Colwill, K. Gladwish, B. Muskat, R. Kinach, S. L. Adams, M. F. Moran, G. B. Morin, T. Topaloglou, and D. Figeys, "Large-scale mapping of human protein–protein interactions by mass spectrometry," *Molecular Systems Biology*, vol. 3, pp. 89 – 89, 2007.

[8] H. Jeong, S. Mason, A.-L. Barabasi, and Z. Oltvai, "Lethality and centrality in protein networks," *Nature*, vol. 411, pp. 41–2, 06 2001.

[9] E. Cho, S. Myers, and J. Leskovec, "Friendship and mobility: User movement in location-based social networks," pp. 1082–1090, 08 2011.

[10] J. Leskovec, J. Kleinberg, and C. Faloutsos, "Graph evolution: Densification and shrinking diameters," *ACM Trans. Knowl. Discov. Data*, vol. 1, mar 2007.

[11] V. Batagelj and A. Mrvar, "Some analyses of erdos collaboration graph," *Social Networks*, vol. 22, no. 2, pp. 173–186, 2000.

[12] C. L. Giles, K. D. Bollacker, and S. Lawrence, "Citeseer: An automatic citation indexing system," in *Proceedings of the Third ACM Conference on Digital Libraries*, (New York, NY, USA), p. 89–98, Association for Computing Machinery, 1998.

[13] L. Šubelj and M. Bajec, "Model of complex networks based on citation dynamics," *WWW 2013 Companion - Proceedings of the 22nd International Conference on World Wide Web*, 03 2013.

[14] J. Yang and J. Leskovec, “Defining and evaluating network communities based on ground-truth,” in *Proceedings of the ACM SIGKDD Workshop on Mining Data Semantics*, (New York, NY, USA), Association for Computing Machinery, 2012.

[15] N. Blagus and M. Bajec, “The network of collaboration: Informatica and uporabna informatika,” *Uporabna Informatika*, vol. 23, no. 1, pp. 22–31, 2015.

[16] B. Rozemberczki and R. Sarkar, “Characteristic functions on graphs: Birds of a feather, from statistical descriptors to parametric models,” in *Proceedings of the 29th ACM International Conference on Information amp; Knowledge Management*, (New York, NY, USA), p. 1325–1334, Association for Computing Machinery, 2020.

[17] L. Subelj and M. Bajec, “Unfolding communities in large complex networks: Combining defensive and offensive label propagation for core extraction,” *Phys. Rev. E*, vol. 83, p. 036103, Mar 2011.

[18] B. Klimt and Y. Yang, “Introducing the enron corpus,” in *CEAS*, 2004.

[19] L. Šubelj and M. Bajec, “Robust network community detection using balanced propagation,” *The European Physical Journal B*, vol. 81, 06 2011.

[20] J. Leskovec and J. Mcauley, “Learning to discover social circles in ego networks,” in *Advances in Neural Information Processing Systems* (F. Pereira, C. Burges, L. Bottou, and K. Weinberger, eds.), vol. 25, Curran Associates, Inc., 2012.

[21] B. Rozemberczki, R. Davies, R. Sarkar, and C. Sutton, “Gemsec: Graph embedding with self clustering,” in *Proceedings of the 2019 IEEE/ACM International Conference on Advances in Social Networks Analysis and Mining*, (New York, NY, USA), p. 65–72, Association for Computing Machinery, 2019.

[22] R. Guimerà, L. Danon, A. Díaz-Guilera, F. Giralt, and A. Arenas, “Self-similar community structure in a network of human interactions,” *Phys. Rev. E*, vol. 68, p. 065103, Dec 2003.

[23] T. Opsahl and P. Panzarasa, “Clustering in weighted networks,” *Social Networks*, vol. 31, no. 2, pp. 155–163, 2009.

[24] N. Eagle and A. (Sandy) Pentland, “Reality mining: Sensing complex social systems,” *Personal Ubiquitous Comput.*, vol. 10, p. 255–268, mar 2006.

[25] “Skitter router adjacencies.” https://catalog.caida.org/details/dataset/skitter_router_adjacencies. Accessed: 25/05/22.

[26] S. Davis, B. Abbasi, S. Shah, S. Telfer, and M. Begon, “Spatial analyses of wildlife contact networks,” *Journal of the Royal Society, Interface / the Royal Society*, vol. 12, 01 2015.

[27] V. Colizza, R. Pastor-Satorras, and A. Vespignani, "Reaction–diffusion processes and metapopulation models in heterogeneous networks," *Nature Physics*, vol. 3, pp. 276–282, 2007.

[28] M. Ripeanu, I. Foster, and A. Iamnitchi, "Mapping the gnutella network: Properties of large-scale peer-to-peer systems and implications for system design," *IEEE Internet Computing Journal*, vol. 6, 10 2002.

[29] C. Song, Z. Qu, N. Blumm, and A.-L. Barabási, "Limits of predictability in human mobility," *Science*, vol. 327, no. 5968, pp. 1018–1021, 2010.

[30] A. Veenstra, "The two blogospheres: Political blog use, participation, and sophistication during the 2008 u.s. election season," *Open Journal of Political Science*, vol. 04, pp. 278–290, 01 2014.

[31] I. S. Duff, R. G. Grimes, and J. G. Lewis, "Users' guide for the harwell-boeing sparse matrix collection (release 1)," *Rutherford Appleton Laboratory Technical Reports*, 1992.

[32] D. J. Watts and S. H. Strogatz, "Collective dynamics of 'small-world' networks," *Nature*, vol. 393, pp. 440–442, 1998.

[33] P. Massa, M. Salvetti, and D. Tomasoni, "Bowling alone and trust decline in social network sites," in *2009 Eighth IEEE International Conference on Dependable, Autonomic and Secure Computing*, pp. 658–663, 2009.

[34] M. Fire, R. Puzis, and Y. Elovici, *Link Prediction in Highly Fractional Data Sets*, pp. 283–300. New York, NY: Springer New York, 2013.

[35] S. Kumar, F. Spezzano, V. S. Subrahmanian, and C. Faloutsos, "Edge weight prediction in weighted signed networks," in *2016 IEEE 16th International Conference on Data Mining (ICDM)*, pp. 221–230, 2016.

[36] J. Leskovec, D. Huttenlocher, and J. Kleinberg, "Signed networks in social media," in *Proceedings of the SIGCHI Conference on Human Factors in Computing Systems*, (New York, NY, USA), p. 1361–1370, Association for Computing Machinery, 2010.

[37] R. Rossi, S. Fahmy, and N. Talukder, "A multi-level approach for evaluating internet topology generators," in *2013 IFIP Networking Conference*, pp. 1–9, 2013.

[38] M. Boguñá, R. Pastor-Satorras, A. Díaz-Guilera, and A. Arenas, "Models of social networks based on social distance attachment," *Phys. Rev. E*, vol. 70, p. 056122, Nov 2004.

[39] N. Spring, R. Mahajan, D. Wetherall, and T. Anderson, "Measuring isp topologies with rocketfuel," *IEEE/ACM Transactions on Networking*, vol. 12, no. 1, pp. 2–16, 2004.

[40] T. Opsahl, "Triadic closure in two-mode networks: Redefining the global and local clustering coefficients," *Social Networks*, vol. 35, no. 2, pp. 159–167, 2013.

[41] D. A. Bader, H. Meyerhenke, P. Sanders, C. Schulz, A. Kappes, and D. Wagner, "Benchmarking for graph clustering and partitioning," in *Encyclopedia of Social Network Analysis and Mining*, 2014.

[42] D. F. Gleich, L. Zhukov, and P. Berkhin, "Fast parallel pagerank: A linear system approach," 2004.

[43] W. De Nooy, A. Mrvar, and V. Batagelj, *Exploratory social network analysis with Pajek*, vol. 27. Cambridge University Press, 2011.

[44] P. Boldi, B. Codenotti, M. Santini, and S. Vigna, "Ubicrawler: a scalable fully distributed web crawler," *Software: Practice and Experience*, vol. 34, no. 8, pp. 711–726, 2004.

[45] C. Castillo, K. Chellapilla, and L. Denoyer, "Web spam challenge 2008," in *AIRWeb 2008*, 2008.
